# Macular Neovascular Membrane as a Late Complication of Acute Posterior Multifocal Placoid Pigment Epitheliopathy (APMPPE) Managed With Intravitreal Aflibercept

**DOI:** 10.1155/crop/3693224

**Published:** 2026-07-14

**Authors:** Ana Paula Couto, Bruno F. Fernandes, Raul N. G. Vianna

**Affiliations:** ^1^ Department of Ophthalmology, Hospital Universitario Antonio Pedro, Fluminense Federal University (UFF), Niteroi, Rio de Janeiro, Brazil, uff.br; ^2^ Argumento Institute, Boucherville, Quebec, Canada

**Keywords:** acute posterior multifocal placoid pigment epitheliopathy, aflibercept, APMPPE, macular neovascular membrane, macular neovascularization

## Abstract

**Purpose:**

To report a case of a macular neovascular membrane (MNV) 14 years after an episode of acute posterior multifocal placoid pigment epitheliopathy (APMPPE).

**Methods:**

Case report.

**Results:**

A 22‐year‐old man presented with acute bilateral blurred vision. The best corrected visual acuity (VA) was 20/200 in both eyes. Fundus examination revealed bilateral multiple yellow‐gray placoid lesions and macular serous detachments. Multimodal imaging and a systemic workup excluded systemic inflammatory or infectious diseases, confirming the diagnosis of APMPPE. The patient was treated with triamcinolone acetonide, an intravitreal injection in the right eye, and a posterior subtenon injection in the left eye. At the 8‐week follow‐up, the fundus lesions healed, with resolution of the macular serous detachment in both eyes. The VA improved to 20/63 bilaterally. After being lost to follow‐up for 14 years, the patient presented sudden worsening of vision in the left eye. The VA decreased to 20/400 in the left eye due to an extensive subretinal hemorrhage and hard exudates in the macula. After diagnostic confirmation of a Type 2 MNV by optical coherence tomography angiography, the patient was treated with three monthly intravitreal injections of aflibercept. The VA improved to 20/160 a month after the treatment.

**Conclusion:**

The treatment with aflibercept in this rare case of MNV secondary to APMPPE effectively controlled membrane activity. However, the fibrosis and atrophy resulting from the MNV contributed to only a partial recovery of VA. Long‐term follow‐up of patients with APMPPE is recommended for early detection and treatment of possible MNV development to improve functional outcomes.

## 1. Introduction

Acute posterior multifocal placoid pigment epitheliopathy (APMPPE) is a rare, self‐limiting inflammatory disorder that primarily affects the retinal pigment epithelium (RPE) and the outer retina, first described by Gass in 1968 [1, 2]. This entity typically affects healthy young adults and is characterized by the sudden onset of bilateral visual disturbances, including blurred vision and scotomas [1]. Fundoscopic examination reveals multiple, flat, creamy‐white placoid lesions at the level of the RPE and choroid, predominantly in the posterior pole, with minimal or absent inflammation in the anterior segment or vitreous [3]. Although the exact etiology remains unclear, APMPPE is believed to be associated with an immune‐mediated process, often triggered by a preceding viral or flu‐like illness. In some cases, systemic inflammatory conditions such as cerebral vasculitis or sarcoidosis may be associated [1]. The lesions generally resolve spontaneously after a single episode, often resulting in a favorable visual prognosis. However, some patients may experience persistent visual impairment depending on lesion location and severity [1, 3].

Macular neovascular membrane (MNV) is a known complication of diseases that affect the choriocapillaris and the RPE. Some placoid‐acquired inflammatory conditions present a higher risk of developing MNV, namely persistent placoid maculopathy (PPM) and serpiginous choroiditis. On the other hand, MNV secondary to APMPPE is rare, with only two cases reported in the literature [3].

## 2. Case Presentation

**Figure 1:**
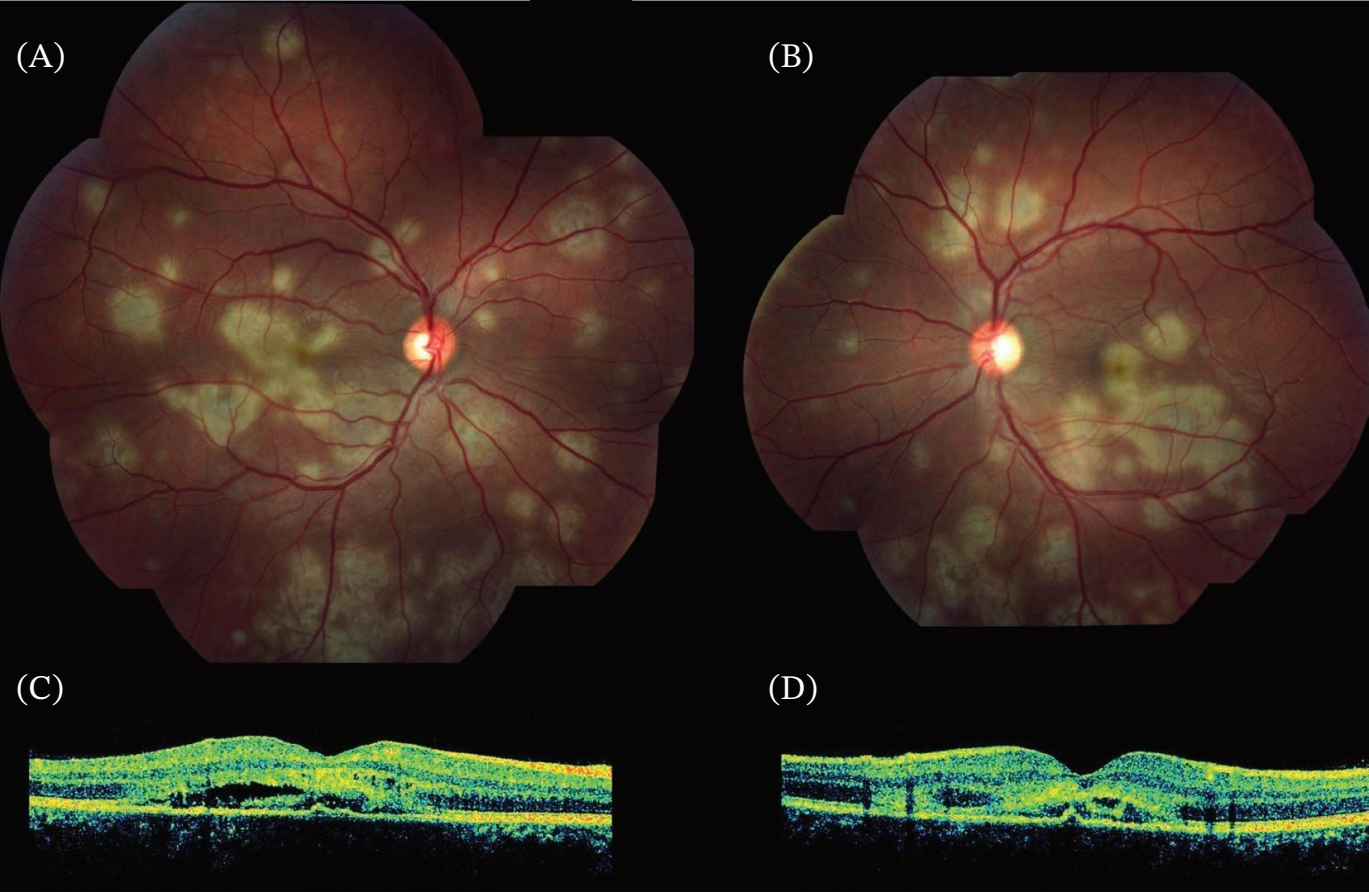
(A and B) Baseline montage color fundus photographs revealed multiple, circumscribed, creamy, yellow‐gray lesions situated in the posterior pole and periphery at the level of the RPE in both eyes. (C and D) Spectral domain‐optical coherence tomography (SD‐OCT) demonstrated macular serous detachment associated with subretinal hyperreflective material in both eyes, as well as bacillary detachment more pronounced in the left eye.

A 22‐year‐old healthy man presented with bilateral painless blurred vision. Past medical and ocular history were unremarkable. The best corrected visual acuity (VA) was 20/200 in both eyes. Slit‐lamp examination of the anterior segment and intraocular pressure were normal. Fundus examination revealed multiple circumscribed, creamy, yellow‐gray placoid lesions, some with geographical borders. The placoid lesions were bilateral, situated at the level of the RPE in the posterior pole and retinal periphery (Figure 1A,B). Spectral domain‐optical coherence tomography (SD‐OCT) revealed bilateral macular serous detachment associated with subretinal hyperreflective material (SHRM) and bacillary detachment (BALAD), with the latter more pronounced in the left eye (Figure 1C,D).

After a systemic workup ruled out inflammatory and infectious diseases, including syphilis, tuberculosis, and dengue fever, the diagnosis of APMPPE was established.

The patient was treated with an intravitreal injection of 4 mg/0.1 mL triamcinolone acetonide in the right eye and a superotemporal subtenonian injection of 40 mg/1 mL triamcinolone acetonide in the left eye. At the 8‐week follow‐up visit, the VA improved to 20/63 bilaterally. Multimodal imaging revealed numerous healed lesions scattered throughout the fundus and the resolution of the macular serous detachment in both eyes.

**Figure 2:**
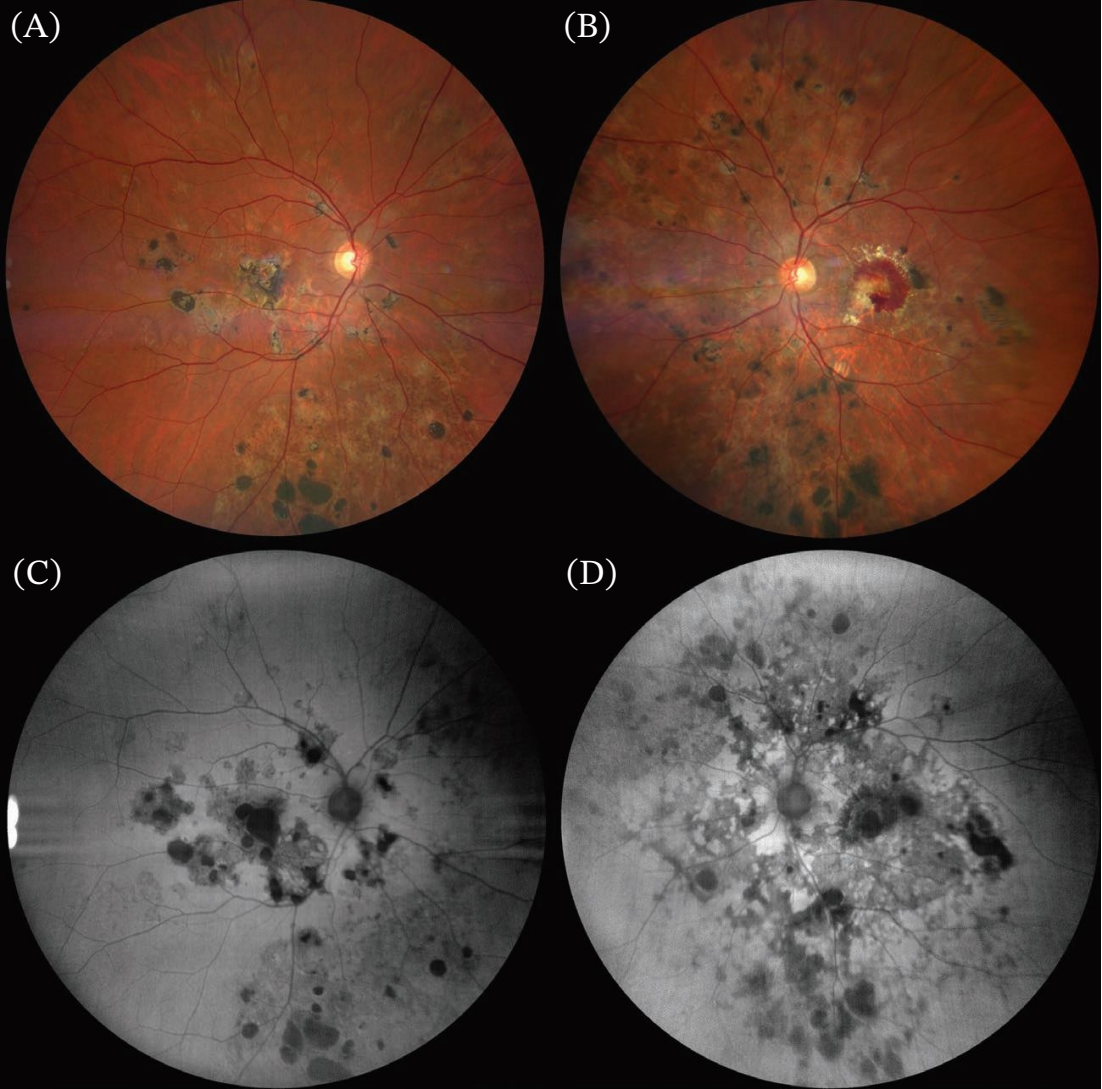
Fourteen years after the initial diagnosis of APMPPE, (A and B) color imaging and (C and D) fundus autofluorescence revealed a (B) significant subretinal hemorrhage associated with hard exudates that developed in the macula of the left eye, along with numerous areas of retinal pigment epithelium (RPE) atrophy and pigment clumps throughout the posterior pole and the retinal periphery in both eyes.

**Figure 3:**
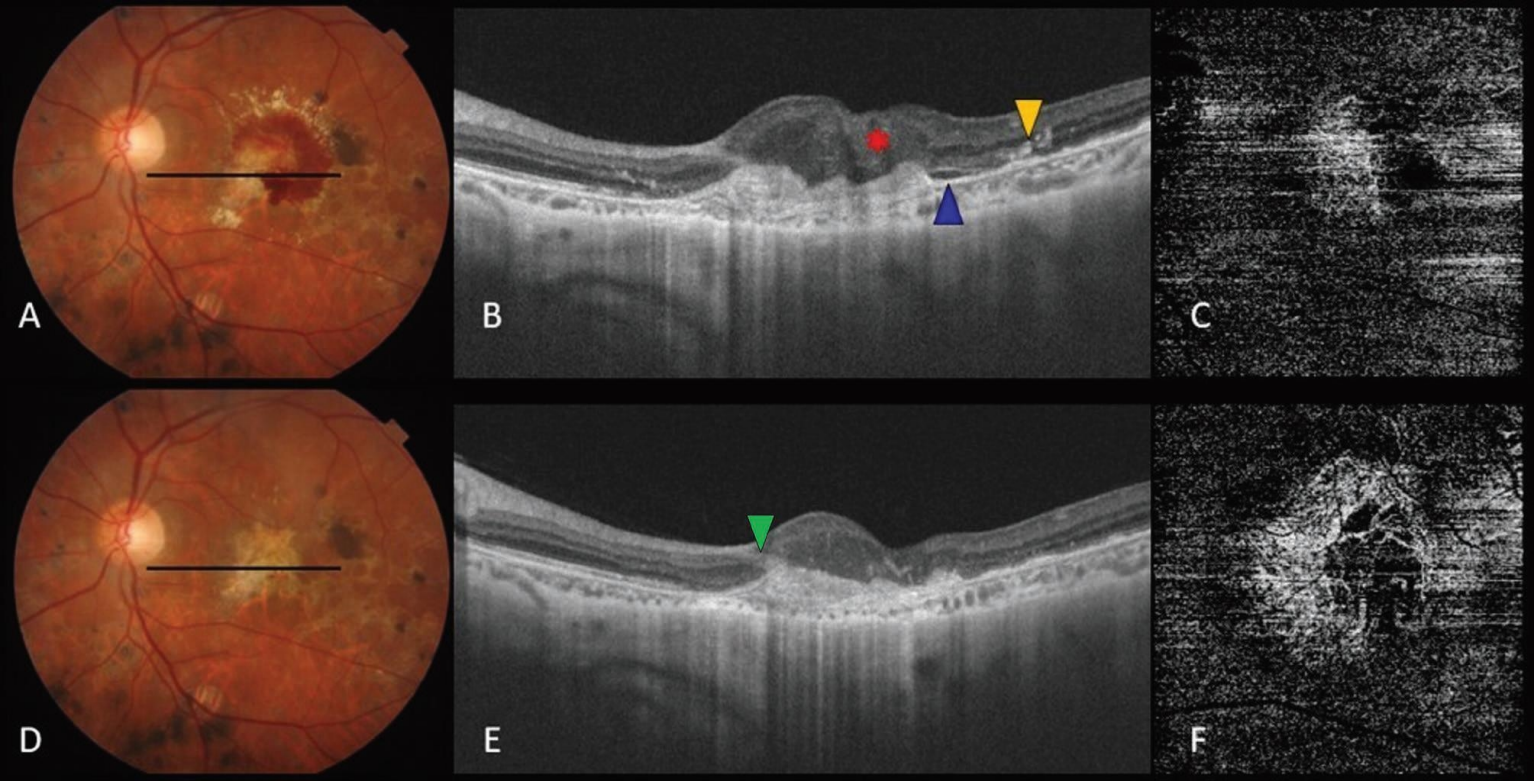
(A–C) Before treatment, spectral domain‐optical coherence tomography (SD‐OCT) of the (B) left eye demonstrated subretinal hyperreflective subfoveal deposits associated with subretinal fluid (blue arrowhead), exudates (yellow arrowhead), and hemorrhage (red asterisk). (C) En face optical coherence tomography angiography (OCTA) scan shows a neovascular net at the level of the outer retina corresponding to the presence of a Type 2 macular neovascular membrane (MNV). (D–F) One month after treatment with intravitreal aflibercept, there was (D and E) an improvement in both exudation and hemorrhage. (E) SD‐OCT displayed a hyperreflective oblique band (HOB) sign extending from the MNV toward the inner retinal layer (green arrowhead). (F) OCTA revealed a larger area of the MNV that was previously obscured by hemorrhage and exudation.

After being lost to follow‐up for 14 years, the patient presented with a sudden worsening of vision in the left eye. The VA was 20/70 in the right eye and 20/400 in the left. Fundus examination of the left eye revealed an extensive subretinal hemorrhage associated with hard exudates in the macula (Figure 2). The SD‐OCT demonstrated increased central retinal thickness and subretinal hyperreflective deposits in the fovea. Optical coherence tomography angiography (OCTA) showed a neovascular net at the level of the outer retina corresponding to a Type 2 MNV (Figure 3A–C).

The left eye was treated with three monthly intravitreal injections of aflibercept. Interestingly, posttreatment OCTA detected a larger neovascularization area previously obscured by the hemorrhage and exudation (Figure 3D–F). One month after the last injection, although the treatment led to anatomical success with resolution of the subretinal fluid and membrane activity, the VA only partially improved to 20/160 in the affected eye due to subretinal fibrosis and atrophy. Quarterly ophthalmologic follow‐up was recommended.

## 3. Discussion

The most frequent symptoms of APMPPE include sudden visual loss accompanied by central or paracentral scotomas [4]. It typically manifests in both eyes within a week, but asymmetrical presentations may occur. The placoid lesions are usually found at the posterior pole of the retina, with involvement of the peripheral retina in some cases [3, 5]. The severity of visual decline is correlated with the location of the lesions, particularly in the case of foveal involvement [3, 4]. The percentage of eyes with full visual recovery is 88%, dropping to only 53% if there is initial foveal involvement [6].

Indeed, SD‐OCT is seminal for the diagnosis and assessing the visual prognosis of inflammatory chorioretinopathies, such as APMPPE, by enabling precise visualization of outer retinal and choriocapillaris alterations. In this case, baseline SD‐OCT was essential in identifying acute APMPPE features, including SHRM and BALAD, indicative of marked exudation and acute photoreceptor disruption [7]. At the final follow‐up, SD‐OCT revealed a hyperreflective oblique band (HOB) nasal to the fovea, extending from the MNV complex toward the inner retina, which resembled the HOB sign recently described in Type 4 MNV associated with age‐related macular degeneration (AMD) [8]. The progression from Type 2 to Type 4 MNV, evidenced by the HOB sign, underscores the dynamic and ischemic nature of the disease. These structural biomarkers provide insight into the underlying pathophysiology and carry significant prognostic implications, as persistent choriocapillaris ischemia and outer retinal damage may predispose to chronic neovascular remodeling and suboptimal visual recovery [9].

Classically, APMPPE is considered a self‐limited disease. The indication of treatment remains controversial since untreated patients have comparable visual outcomes to those who received treatment [1, 5]. Moreover, patients are often referred to specialists late in the course of the disease when APMPPE lesions are already healing [3]. Conversely, the rationale for the indication for treatment includes the location of lesions in the macula, presence of subretinal fluid, and significant visual impairment [5]. Despite limited evidence in the literature, the most common APMPPE treatment includes oral, intravenous, intravitreal, or subtenon corticosteroids [3, 5]. In our patient, local corticosteroid administration was chosen due to socioeconomic constraints and concerns regarding systemic therapy. During follow‐up, no steroid‐related complications, such as ocular hypertension or cataract progression, were observed.

Macular neovascular membrane (MNV) may develop secondary to APMPPE regardless of previous therapy [3, 4]. Bowie et al. [10] reported a case of MNV 29 years after the onset of APMPPE. The patient had a favorable outcome with subtenon triamcinolone injection and photodynamic therapy (PDT) despite potential collateral damage of the PDT, with the VA improving from 20/100 to 20/25. Mavrakanas et al. [11] reported an MNV that developed 2 years after the initial diagnosis of APMPPE. The MNV was treated with a single intravitreal injection of ranibizumab, and the VA recovered from 20/40 to 20/20. However, the characteristics of the bilateral lesions, along with the findings from fluorescein angiography and optical coherence tomography (OCT), were atypical for APMPPE and were more consistent with idiopathic or acquired vitelliform disease.

There is a consensus that the development of MNV as a complication of placoid‐acquired inflammatory diseases should be treated with intravitreal injections of antivascular endothelial growth factor (VEGF) [3, 12]. In PPM, MNV can occur in approximately 50% of eyes, and in serpiginous choroiditis, the risk is considerably higher if there is macular involvement. On the other hand, MNV is a rare complication of APMPPE [1, 3]. Treating MNV secondary to placoid diseases with intravitreal anti‐VEGF generally leads to a decrease in exudation, inducing MNV regression with favorable outcomes. However, in some cases, visual impairment may result from subsequent fibrosis or atrophy [3]. Patients should be informed about this potential complication and monitored closely during long‐term follow‐up. The typical patient profile may contribute to delayed detection of MNV secondary to APMPPE, as this disease usually affects young individuals who often experience substantial visual recovery after the acute episode. Consequently, many patients are discharged from regular ophthalmologic care or become lost to follow‐up. This may create a diagnostic gap in which late‐onset complications, such as MNV, remain undetected until they become symptomatic.

In the case presented herein, the patient was lost to clinical follow‐up and remained asymptomatic for 14 years. Follow‐up visits during this asymptomatic period would have enabled multimodal imaging to detect a subclinical MNV and monitor any signs of exudation before the onset of symptoms. Timely intervention in cases of disease activity increases the chances of preventing extensive subretinal scarring and atrophy that can impact full functional recovery.

In conclusion, MNV is a rare vision‐threatening complication of APMPPE. Close follow‐up of patients following an episode of APMPPE, including noninvasive imaging methods such as OCT and OCTA, is recommended for early detection of MNV development and timely therapeutic interventions. Aflibercept treatment effectively controlled MNV activity in this rare case secondary to APMPPE. Although treatment with anti‐VEGF can effectively control exudation, early treatment, ideally in the subclinical phase, is more likely to yield more favorable visual outcomes.

## Author Contributions

All authors attest that they met the current ICMJE criteria. Data collection and interpretation were conducted by A.P.C. and R.N.G.V. Figures were prepared by A.P.C., R.N.G.V., and B.F.F., A.P.C. and B.F.F. were major contributors in writing the manuscript. R.N.G.V. provided critical revisions.

## Funding

No funding was received for this manuscript.

## Disclosure

All authors read, reviewed, and approved the final manuscript.

## Consent

Informed consent was obtained from the patient in writing.

## Conflicts of Interest

The authors declare no conflicts of interest.

## Data Availability

The data that support the findings of this study are available from the corresponding author upon reasonable request.
